# *Trypanosoma cruzi*, Etiological Agent of Chagas Disease, Is Virulent to Its Triatomine Vector *Rhodnius prolixus* in a Temperature-Dependent Manner

**DOI:** 10.1371/journal.pntd.0003646

**Published:** 2015-03-20

**Authors:** Simon L. Elliot, Juliana de O. Rodrigues, Marcelo G. Lorenzo, Olindo A. Martins-Filho, Alessandra A. Guarneri

**Affiliations:** 1 Department of Entomology, Universidade Federal de Viçosa, Campus Universitário, Viçosa, Minas Gerais, Brazil; 2 Centro de Pesquisa René Rachou, Belo Horizonte, Minas Gerais, Brazil; Lancaster University, UNITED KINGDOM

## Abstract

It is often assumed that parasites are not virulent to their vectors. Nevertheless, parasites commonly exploit their vectors (nutritionally for example) so these can be considered a form of host. *Trypanosoma cruzi*, a protozoan found in mammals and triatomine bugs in the Americas, is the etiological agent of Chagas disease that affects man and domestic animals. While it has long been considered avirulent to its vectors, a few reports have indicated that it can affect triatomine fecundity. We tested whether infection imposed a temperature-dependent cost on triatomine fitness. We held infected insects at four temperatures between 21 and 30°C and measured *T*. *cruzi* growth *in vitro* at the same temperatures in parallel. *Trypanosoma cruzi* infection caused a considerable delay in the time the insects took to moult (against a background effect of temperature accelerating moult irrespective of infection status). *Trypanosoma cruzi* also reduced the insects’ survival, but only at the intermediate temperatures of 24 and 27°C (against a background of increased mortality with increasing temperatures). Meanwhile, *in vitro* growth of *T*. *cruzi* increased with temperature. Our results demonstrate virulence of a protozoan agent of human disease to its insect vector under these conditions. It is of particular note that parasite-induced mortality was greatest over the range of temperatures normally preferred by these insects, probably implying adaptation of the parasite to perform well at these temperatures. Therefore we propose that triggering this delay in moulting is adaptive for the parasites, as it will delay the next bloodmeal taken by the bug, thus allowing the parasites time to develop and reach the insect rectum in order to make transmission to a new vertebrate host possible.

## Author Summary

Parasites are often assumed to cause little harm to their arthropod vectors, even though they commonly reproduce inside the arthropods and exploit their nutrients, even causing lesions when crossing internal barriers. Thus, the interests of parasite and vector may well not be aligned and we can expect the parasite to exploit its vector just as it does with its main host, with consequent negative effects on the vector’s fitness. Here, we show that this occurs with *Trypanosoma cruzi* in its bug vector (*T*. *cruzi* causes Chagas disease, affecting ca. 8 million people and disease management is principally attained via vector control). Our results indicate that the parasites delay insect moulting, which is likely beneficial to them as they need time to develop in the insect before the next bloodmeal (that only occurs post-moult). We also show parasite-induced mortality over the narrow range of temperatures which the insect prefers and over which it performs best. *In vitro* growth of the parasite increases with temperature and we discuss how this may help explain the effects *in vivo*. Overall, these results will be important to understand the epidemiology of Chagas disease and provide an evolutionary context to explain the parasite′s interaction with its vector.

## Introduction

A long-standing implicit assumption in the literature on vector-borne diseases is that the parasite does little or no harm to its vector (see [1] for a review). This makes considerable intuitive sense as the parasite relies on the vector for its transmission, so negative effects on the vectors’ fitness could be expected to reflect negatively on the parasites’ fitness. This was perhaps best formulated (verbally rather than mathematically) in Ewald’s classic treatise on the evolution of virulence [2]. With the rapid development of theory on the evolution of virulence in recent years [3], it has become clear that the vector should to a large degree be considered an alternative host for the parasite, one in which a certain degree of host exploitation (and consequent virulence to this ‘host’) is to be expected [1]. Meanwhile, empirical studies that are aimed at detecting fitness effects of parasite infection have become more refined, looking beyond fecundity and mortality to hunt for more subtle life history or behavioral effects, for example. This can be seen particularly in studies of mosquito (Culicidae) infection with pathogens, such as negative effects of dengue virus on fecundity and oviposition success in *Aedes* [4]. Perhaps the most elegant demonstration that the interests of parasite and vector are not entirely aligned is parasite-induced increases in biting rates in mosquitoes [5–9], sand flies [10] and tsetse flies [11,12]—this is likely to increase transmission (and thereby fitness) of the parasite while the vector is liable to suffer a reduction in fitness due to excessive energy expenditure and increased risk of mortality when attempting to bite. Meanwhile, evidence of an interplay between parasite and vector strategies towards one another can be seen in the case of several parasites of plants that are transmitted by insect vectors. In several systems where parasite and vector are believed to have shared a coevolutionary history, the parasite increases its vector’s fitness indirectly via effects on the host plant (e.g. [13,14]). This positive interaction is illustrative as the vector will likely spend several generations on the main host (the plant), a situation very different from most vectors of parasite diseases of humans that interact only briefly with the main hosts and in which negative effects can be expected.

For vector-borne diseases of humans, such considerations are of great importance for vector management, especially when novel technologies are under consideration. In strategies such as the release of transgenic vectors, paratransgenesis or use of biocontrol agents that interfere with transmission, the life history and behavior of the vector are key factors [15,16], as are possible evolutionary responses of vector and parasite [17]. It is vital, then, to understand how vector and parasite interact in terms of their respective fitnesses and possible patterns of selection. Chagas disease is one such example. *Trypanosoma cruzi* is a digenetic protozoan that infects mammals and triatomines in the Americas. As a result of anthropic activities this enzootic infection affects man and domestic animals, causing to the first a disease with different levels of pathology. As a comparatively recently described disease (Chagas disease was first described by Carlos Chagas in 1909) research has focused on interactions between the parasite and man, with little consideration of parasite effects on the invertebrate hosts. Further, as earlier studies showed no parasite-induced alterations in triatomine physiology [18], the parasite has long been considered avirulent to its vectors [19–21]. Few studies showing alterations on fecundity rates of infected females have been conducted [22, 23]. Furthermore, our group has recently shown that *T*. *cruzi* affects fecundity and fertility rates of *R*. *prolixus* depending on the temperature at which insects are raised [24].

We sought, then, to investigate how *T*. *cruzi* might affect its triatomine hosts. As the parasite does not invade the insects’ body but develops rather in its intestine, we might expect effects on the insects’ fitness to be marginal. Previous studies showed no effect of *T*. *cruzi* on the development of *Nocardia sp*. and *Rhodococcus rhodnii*, gut symbionts of *Triatoma infestans* and *Rhodnius prolixus*, respectively [25]. However, as a consequence of living only in the insect intestinal tract, *T*. *cruzi* probably competes with its host for nutritional resources. In addition, most laboratory studies of *T*. *cruzi*-triatomine interactions have evaluated fitness parameters under conditions that aim to maximize vector development and survival. Changes in mortality rates in mosquitoes under glucose deprivation have been demonstrated for *Plasmodium* [26,27] and dengue virus infections [4]. Therefore, our prediction is that the parasite might have negative effects on its host’s fitness under less than optimal (and therefore more realistic) environmental conditions [28].

In addition, temperature is a factor of particular importance in host-parasite interactions, especially when the host is ectothermic. It can be a key factor in determining whether a host-parasite interaction eventually favors host or parasite, while in some instances the nature of the interactions can only really be understood by observing the host-parasite interaction under different thermal conditions [29, 30]. We therefore chose to conduct our study under four thermal regimes, and to use a comparatively narrow range of temperatures to keep the test conservative.

## Materials and Methods

### Ethics statement

All experiments using live animals were performed in accordance with FIOCRUZ guidelines on animal experimentation and were approved by the Ethics Committee in Animal Experimentation (CEUA/FIOCRUZ) under the approved protocol number L-058/08. The protocol is from CONCEA/MCT (http://www.cobea.org.br/), which is associated with the American Association for Animal Science (AAAS), the Federation of European Laboratory Animal Science Associations (FELASA), the International Council for Animal Science (ICLAS) and the Association for Assessment and Accreditation of Laboratory Animal Care International (AAALAC).

### Insects and parasites

*Rhodnius prolixus* used in assays were obtained from a laboratory colony which is derived from insects collected in Honduras around 1990. The colony was maintained by the Vector Behaviour and Pathogen Interaction Group in Centro de Pesquisas René Rachou, FIOCRUZ, Brazil. Insects were reared at 26 ± 1°C and relative humidity of 65 ± 10%, with natural illumination. They were fed on chicken and mice anesthetized with an intraperitoneal injection of a ketamine (150 mg/kg; Cristália, Brazil)/xylazine (10 mg/kg; Bayer, Brazil) mixture. When insects were infected, they were fed on an artificial feeder containing a suspension of freshly collected and inactivated human blood (56°C/30min) [24], using standard aseptic procedures. Therefore, the parasites did not enter into contact with the anesthetic mixture. Note also that as a routine procedure, *T*. *cruzi* cultures were checked for bacterial contamination in every passage under the microscope. Therefore, these procedures assured no bacterial contamination in the blood or *T*. *cruzi* cultures.

The *T*. *cruzi* used was ‘CL’ strain, originally isolated from naturally-infected *T*. *infestans* from southern Brazil [31] and subsequently kept in laboratory cultures. Epimastigote forms were cultured at 27°C in liver infusion tryptose (LIT) medium supplemented with 15% fetal bovine serum, 100mg/ml streptomycin and 100units/ml penicillin. Parasite passages were performed twice a week, i.e. ca. once every three days. As it has been shown that trypanosomes tend to lose infectivity if they are not frequently exposed to hosts [32], parasites were passed through mice and triatomines every 6 months. Briefly, 5^th^ instar nymphs were infected with culture epimastigotes through artificial feeding. One month after infection, these insects were fed and their urine containing metacyclic trypomastigotes was collected and inoculated into a Swiss mouse. Two weeks after inoculation the parasites were recovered by cardiac puncture and used to perform a hemoculture. For infection assays, 50–100μl of culture were washed in sterile PBS (0.15M NaCl, 0.01M sodium phosphate, pH 7.4; 2,000 RPM) and resuspended in a final volume of 50μl.

### Temperature effects on infected insects

Seven day old second instar nymphs (n = 126) were fed on a suspension of freshly collected and inactivated human blood (56°C/30min) with culture epimastigotes. Aiming to prepare 5ml of inoculum at 1x10^7^ parasites/ml of blood, we took a volume of culture that would give us 5x10^7^ parasites total. This volume of culture was washed in PBS, centrifuged and resuspended in 50μl of PBS. This was then added to 5ml of blood. Since a second instar nymph ingests between 20–24μl of blood, we estimated that each one ingested approximately 200,000 parasites. Insects used for the control group were fed on the same inactivated blood at the same conditions, except for the parasite presence (n = 132). One day after feeding, the insects were transferred to Petri dishes whose bases were lined with filter paper discs (up to seven insects per plate; 5 plates/treatment). These were placed in temperature control chambers at 21±0.2, 24±0.2, 27±0.2 or 30±0.2°C and no further food was offered during the experiment. The times taken to reach third instar and mortality rates were recorded up to 90 days after the first moult. Insect mortalities were recorded for both treatments at 30, 60 and 90 days after ecdysis to the third instar. The entire intestinal tracts of infected insects—dead or alive at the end of the experiment—were macerated and examined to confirm parasite infection.

### Temperature effects on viability and *in vitro* growth of *Trypanosoma cruzi*

Culture epimastigotes were transferred at an initial concentration of 1x10^6^/ml to cell culture flasks (25cm^2^) containing fresh LIT medium to a final volume of 8ml. The flasks were immediately transferred to four independent controlled temperature chambers (21±0.2, 24±0.2, 27±0.2 and 30±0.2°C) and kept there for seven days. Two replicate culture samples were simultaneously tested for each temperature. Daily, a 50μl sample was collected from each flask and stained for flow cytometry absolute counts and viability analysis.

Dual label fluorescent staining procedures were performed per sample to determine the absolute counts of live and dead parasites in each sample. For this purpose, 50μl of culture were incubated in the presence of 120μl of PBS and 25μl of fluorescein diacetate (FDA) at 7μg/ml plus 5μl of propidium iodide (PI) at 25μg/ml, both from Sigma (St Louis, MO, USA) for 10 min at room temperature. FDA (Sigma 7378) stock solution was prepared at 1mg/ml in acetone and stored at −20°C until use. PI stock solution was prepared in ddH_2_O at 1 mg/ml and stored at −20°C until use.

Following incubation, 20μl of fluorosphere suspension were added to each tube immediately before flow cytometric acquisition. As many flow cytometers cannot directly provide the cell concentration or absolute count of cells in a sample, the Flow-Count Fluorosphere (lot #7548025 bead counts of 986 beads/μl, Beckman Coulter, Inc., Miami Lakes, FL, USA) were used as a calibration device to directly obtain absolute counts of parasites using flow cytometry.

Quantitative flow cytometric double labeling assay, calibrated with fluorospheres, was used to simultaneously determine the number of parasites along the growth curve, as well as to calculate the mortality rate. In order to obtain the number of total epimastigotes/μL of LIT cultures, following flow cytometer acquisition of approximately 5,000 fluorospheres per sample, data analysis was carried out as follows: A bidimensional pseudocolor graph of granularity (SSC) *versus* non-related fluorescence 3 chart was created to exclude autofluorescent (FL3 positive) events outside the region R1. Following this, the events inside the R1 were displayed on size *versus* granularity plots to select and quantify the bead cluster (BEADS) and epimastigote population (EPI) as illustrated in S1 Fig. EPI gated events were then analyzed further on FL1 (FDA) *versus* FL2 (PI) charts to quantify the frequency of PI+FDA positive events (DEAD EPI = MORTALITY RATE) as well as FDA single positive cells (LIVE EPI) (S1 Fig). The calculation of the final concentration of TOTAL EPI and the VIABLE EPI counts were achieved with the following equations:

$$Totalepi=\frac{epi}{50\times beads÷19,720}$$

where EPI = number of epimastigote event counts for a given tube, 50 = volume of culture suspension added to each tube; BEADS = number of fluorosphere beads aspirated in a given tube and 19,720 = number of fluorosphere beads added to each tube, considering the volume of 20ml of bead suspension.

$$Viableepi=\frac{totalepi\times liveepi}{100}$$

where liveepi = percentage of FDA single positive events and 100 = the percentage conversion factor.

A dye-free sample was used as a control. A FACScan Becton Dickinson flow cytometer (La Jolla, CA, USA) was used for acquisition and the FlowJo software 9.6.3 (San Diego, CA, USA) used for data analysis using pseudocolor charts. Representative flow cytometry charts are provided in the figures.

### Statistical analyses

The times that infected and uninfected insects took to die were estimated using Kaplan—Meier survival analyses. Comparisons were made with log-rank tests. Intermoult periods were compared through a nested ANOVA with Petri dish groups nested within both feeding and infection status. As no significant differences were found among the Petri dishes (F = 1.178, p = 0.286) *post hoc* comparisons (Tukey HSD test) were performed adding all data of the five groups of the respective temperatures.

Analyses of temperature effects on *in vitro* parasite growth were conducted in R version 2.13.0 [33]. The first step was to determine growth rates (i.e. regression slopes) for each replicate (bottle) for each temperature treatment. For this, live parasite population sizes were log-transformed (i.e. log_10_ of parasite number +1) and linear mixed effects models were used to account for the repeated measures (i.e. days 1, 2, 3 and so on). Eight growth rate values were therefore obtained (two replicates x four temperatures). These were subjected to regression analyses aimed at detecting temperature effects on growth rates, in particular, to test whether growth rates could be seen to peak at different temperatures.

## Results

### Development of infected nymphs at different temperatures

**Fig 1:**
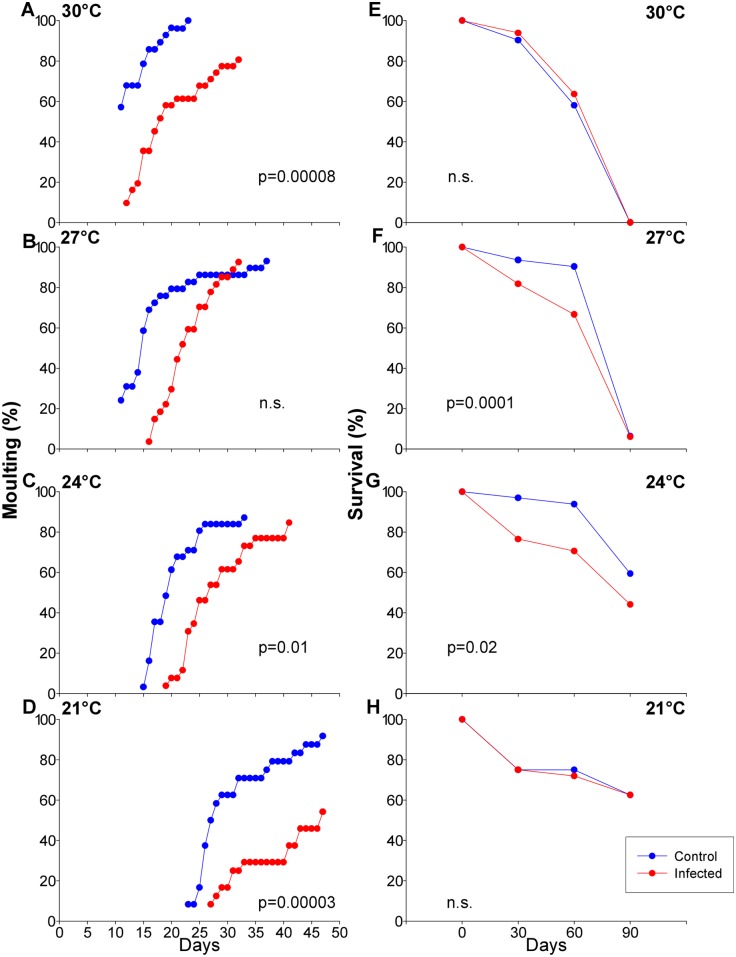
Effects of *Trypanosoma cruzi* infection on second instar *Rhodnius prolixus* nymphs over four temperatures. Insects were fed a blood meal at day 0 and were offered no further food. (A-D) time required to moult from second to third instar. (E-H) survival at 30, 60 and 90 days post-blood meal. P values indicated in each graph represent statistical significances of comparisons of infected versus uninfected control insects, using Tukey HSD *post hoc* tests from nested ANOVA analyses.

The time taken to moult from second to third instar was affected by both infection (nested ANOVA, F = 67.445, p = 0.00001) and temperature (nested ANOVA, F = 68.967, p = 0.00001). The period was reduced by increasing temperatures up to one third for uninfected insects and half for infected insects (Fig. 1A-D). Meanwhile, infection with *T*. *cruzi* delayed moult by 6–11 days (Fig. 1A-D, Tukey HDS; 32.1±8.3 (control) *vs*. 43.5±9.2 (infected) days for 21°C (p = 0.00003), 23.2±9.0 (control) *vs*. 30.0±7.7 (infected) days for 24°C (p = 0.017), 17.8±8.9 (control) *vs*. 23.6±6.3 (infected) days for 27°C (p = 0.08), and 13.3±3.2 (control) *vs*. 23.3±10.4 (infected) days for 30°C (p = 0.00008)).

At the lowest temperature (21°C), mortality in uninfected control insects was more than 20% after 30 days (Fig. 1H). This initial mortality of uninfected insects was much reduced at the higher temperatures but by the end of the observations (90 days), these uninfected insects had almost all died at the higher temperatures. Against this background, infection with *T*. *cruzi* was found to accelerate mortality in the two intermediate temperatures, 24 (P = 0.02) and 27°C (P = 0.0001) (Fig. 1F & G), but not at 21 or 30°C (Fig. 1E & H).

### Temperature effects on *in vitro* growth of parasites

**Fig 2:**
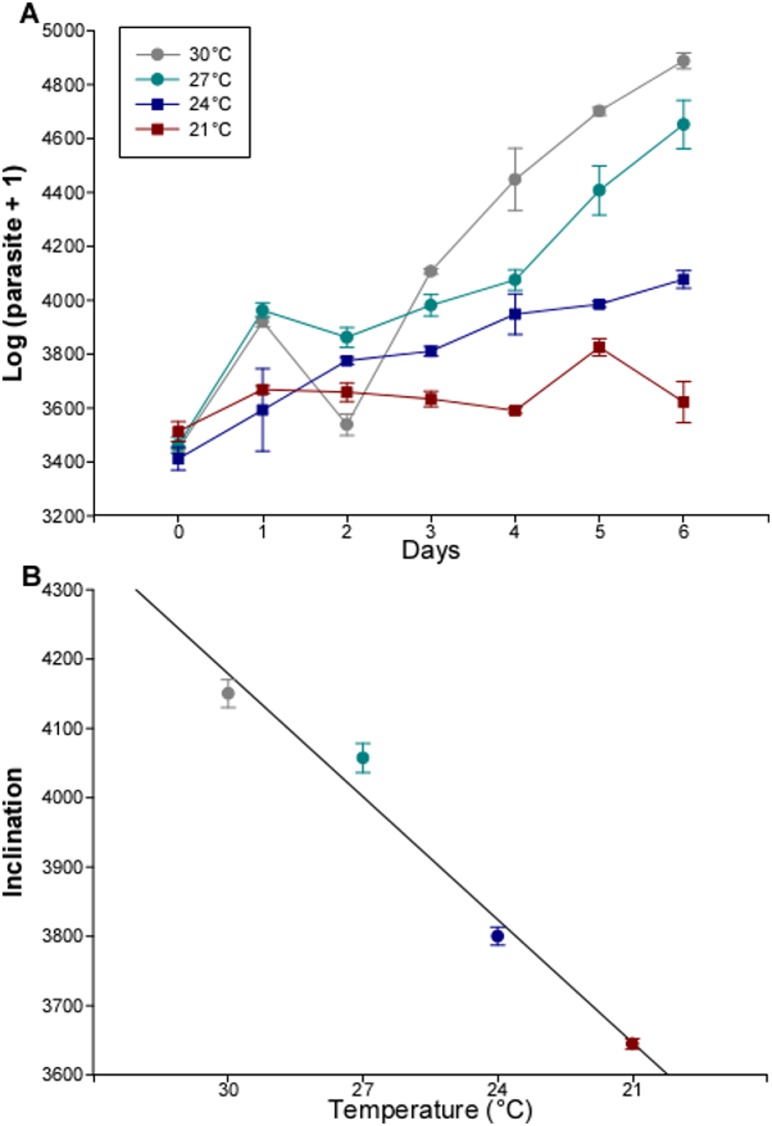
Growth of cultures of *Trypanosoma cruzi* epimastigotes kept under different temperatures. (A) growth through time (live parasites counted in a flow cytometer after staining with fluorescein diacetate). (B) growth rates with temperature. Error bars are standard errors obtained from two replicates.

The population growth of *T*. *cruzi in vitro* increased consistently with increasing temperature (Fig. 2; p<0.0001 for temperature effect). The best-fit regression of growth rates against temperature (Fig. 2B) was not curved, so peak growth would have occurred at or above 30°C.

Fluorescein diacetate-propidium iodide staining made it possible to distinguish live cells from those that had already started to die, these last being stained by both dyes (S1 Fig). Mortality rates of *T*. *cruzi* were below 5% at 27 and 30°C, reaching ca. 15% at 24°C and over 20% at 21°C (S1 Fig).

## Discussion

To the best of our knowledge, the present study is the first report of temperature-modulated mortality caused by a protozoan parasite of medical importance to its arthropod vector. In the case of malaria-mosquito systems, decreased mosquito survival during infection is only seen in unnatural combinations, although natural combinations exhibit a tendency towards such increases in mortality [34]. More recently, a natural combination of *Plasmodium*-mosquito (in this case an avian malaria system), showed an increase in longevity associated with a decrease in fecundity in infected mosquitoes [35]. It is now well-established that arboviruses can be virulent to their culicid vectors, depending on the taxonomic groups and the mode of virus transmission [36]. In dengue virus-*Aedes* associations it has been observed that the virus presence affected several mosquito fitness parameters such as survival, fecundity and oviposition success [4].

This accelerated mortality of *R*. *prolixus* infected with *T*. *cruzi*, under conditions of starvation (commonly experienced by these insects—[28]), was seen over a narrow range of temperatures (i.e., at 24 and 27°C but not at 21 or 30°C). High temperatures associated with prolonged starvation were lethal to insects, independently of parasite infection. It is well known that high temperatures promote an increase in the metabolism of insects (reviewed by [37]). Therefore, an increased mortality would be expected in starved insects submitted to higher temperatures, as already seen in previous studies [38]. According to the effect of temperature on *T*. *cruzi* growth in culture media, the mortality of infected insects would be expected to occur as a consequence of large parasite populations developed at higher temperatures. Nevertheless, there were no differences in mortality rates between infected and healthy insects kept at 30°C. This was probably a result of a lack of nutritional resources for parasite development in starved insects. In fact, it has already been demonstrated that triatomines can eliminate *T*. *cruzi* infections after long periods of starvation [39]. Curiously, *R*. *prolixus* prefers temperatures of 25.0–25.4°C when offered a choice and performs best around these temperatures [40]. Furthermore, temperatures in the sylvatic ecotopes in which this insect is to be found oscillate closely around 25°C [41]. While we might have expected the vector to be less affected by the parasite at temperatures near to its optimum (as is the case with locusts infected with the fungus *Metarhizium anisopliae*, [29]) we might also expect the parasite to be adapted to perform optimally at exactly these temperatures. If this is the case, then we must conclude that *T*. *cruzi*’s strategy, in its vector, results in direct physiological harm to its vector, that can be observed as vector mortality. At this range of temperatures, the parasite has a high *in vitro* growth rate (Fig. 2) so we hypothesize that its strategy in the vector is close to unrestrained growth, trading off an increased chance of transmission (due to a high population density in the intestine) with the cost of killing its vector and so effecting zero transmission. Given these insects are able to display temperature preferences [40, 42–45], we might expect them, when infected with *T*. *cruzi*, to alter their thermal preferences. All of these factors are liable to affect *T*. *cruzi* transmission dynamics and ultimately, epidemiology.

The number of *T*. *cruzi* parasites increased in direct relation to temperature. In fact, after seven days, parasites kept at 30°C increased their numbers close to 28 times, almost doubling their growth rate at 27°C. Previous studies have shown that both *T*. *cruzi* epimastigote and trypomastigote forms grow when exposed to 37°C [46]. The lowest temperature tested here seemed to have a harmful effect on *T*. *cruzi*, since their mortality at this temperature was close to 20%. It has been suggested that low temperatures affect the endocytic processes in *T*. *cruzi* epimastigotes [47]. Low-temperature blockage of endocytosis has also been reported in many eukaryotic cells [48–51]. Whether these effects of low temperature on parasite endocytosis are related to the poor performance of *T*. *cruzi* at 21°C deserves to be analyzed in future experiments. Temperature is important in the development and within-host dynamics of several other protozoan parasites. *Leishmania* species differ in their susceptibility to temperature stress, as reflected in their ability to establish infections at different sites in the mammalian body [52]. The temperature resistance of *Leishmania* spp. has been related with the parasite tropism, as visceral species are more temperature resistant than cutaneous species [53]. Temperature has also been shown to be important to regulate the membrane potential across the plasma membrane and the internal pH in *Trypanosoma brucei* [54]. In addition, the reduction in temperature from 37 to 27°C and the addition of cis-aconitate are enough to trigger the transformation of the monomorphic *T*. *brucei* from bloodstream to procyclic trypomastigotes in culture medium [55].

Beyond mortality, infection with *T*. *cruzi* considerably delayed moult in *R*. *prolixus*, across the range of temperatures tested. In contrast to results observed with other triatomine species [18–22], moulting in *R*. *prolixus* second instar nymphs was delayed by more than 10 days in a single developmental stage. In an entire life cycle the accumulation of this effect could possibly prolong by more than a month the time needed to reach the adult stage. It is highly likely that such a delay would affect insect fitness. While it will be interesting to look for physiological explanations for this (there is some evidence indicating a possible competition for lipids in this host-parasite system [56]), there may be a very good adaptive explanation, in terms of the parasite’s fitness. *Trypanosoma cruzi* has been reported to take approximately up to month at 28°C to colonize the intestine, reach the rectum and differentiate into infective stages [57, 58]. As triatomines will only feed again after they have moulted, it would benefit the parasite if their moult, and thus the next bloodmeal, were delayed until such a time as the parasite is in the right place and life stage to be transmitted to a vertebrate host. Such a delay would then favor parasite transmission.

Effects of *T*. *cruzi* infections on triatomine fitness have previously been described in the literature. Schaub and Lösch [59] observed that the resistance of infected *T*. *infestans* was reduced when insects were starved. However, in subsequent studies from the same group the parasite was considered subpathogenic to its invertebrate hosts since, apparently, it does not damage the vector under optimal conditions [60,61]. Meanwhile, Botto-Mahan [62] evaluated the time to moult during the ontogeny of *Mepraia spinolai* infected by *T*. *cruzi* (kept at 26°C) and showed that infected insects presented a delayed moult and an increased mortality when compared with control ones. However, insects from the infection treatment were always fed on infected mice, and as mentioned by the author, it is not possible to be sure that the observed effects were not a result of differences in blood quality between infected and non infected mice. Nevertheless, the deleterious effects of *T*. *cruzi* described in these studies altogether with the results presented in this study and the alteration of the reproductive fitness of *R*. *prolixus* induced by *T*. *cruzi* recently demonstrated by our group [24] represent a bulk of evidence confirming fitness costs induced by this parasite.

To conclude, we have shown that the medically-important parasite *T*. *cruzi* can exert virulence effects on the vector *R*. *prolixus*. This effect is strongest over exactly the temperature range preferred by the insect and in which it is to be found in the wild (often infected with *T*. *cruzi*). The ability of *T*. *cruzi* to develop over a broad temperature range might have contributed to its adaptation to a larger number of triatomines. It will be important to investigate virulence effects in other vector species, behavioural responses of the insects to infection (see [63] for example) and impacts on transmission dynamics.

## Supporting Information

S1 Fig Impact of temperature on the growth rate and viability of *Trypanosoma cruzi* epimastigotes. Dual-color flow cytometry (FDA+PI) using fluorescent calibration beads to determine absolute epimastigote counts (line charts) and mortality (bar charts). The latter was calculated considering PI positive and PI+FDA double positive events (PI+FDA stained parasites) detected in cultures exposed to different temperatures (●21°C, ◯24°C, ▾ 27°C, ▿ 30°C). (A) Parasite growth curves express the number of epimastigotes/μl and the corresponding mortality is indicated by bars. (B) Representative flow cytometry pseudocolor charts are provided to illustrate the morphometric profile (Forward Scatter—Size *vs* Side Scatter—Granularity) and fluorescent pattern observed at control samples (incubated in the presence of PBS) as well as PI+FDA stained parasites and FDA single positive viable parasites.
(TIFF)
