# Supplementary figures and images for: Trypanosoma cruzi, Etiological Agent of Chagas Disease, Is Virulent to Its Triatomine Vector Rhodnius prolixus in a Temperature-Dependent Manner
Source: PLoS Negl Trop Dis. 2015 Mar 20;9(3):e0003646. doi: 10.1371/journal.pntd.0003646 (PMC4368190; doi:10.1371/journal.pntd.0003646)

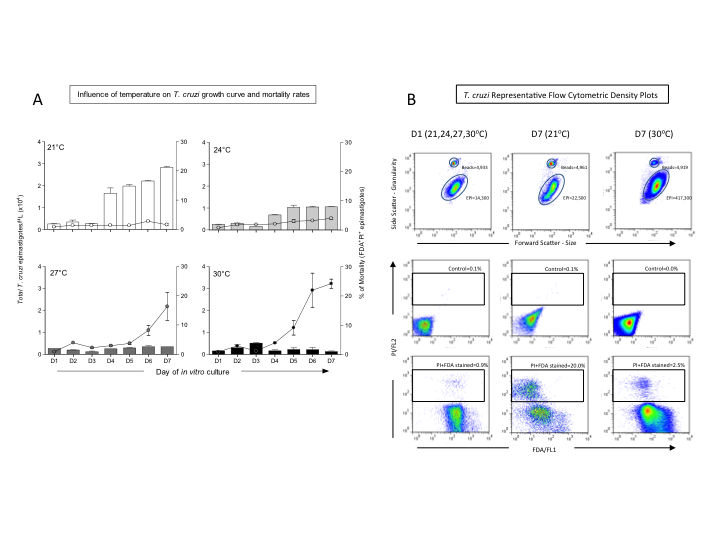

Supplement: S1 Fig — Dual-color flow cytometry (FDA+PI) using fluorescent calibration beads to determine absolute epimastigote counts (line charts) and mortality (bar charts). The latter was calculated considering PI positive and PI+FDA double positive events (PI+FDA stained parasites) detected in cultures exposed to different temperatures (●21°C, ◯24°C, ▾ 27°C, ▿ 30°C). (A) Parasite growth curves express the number of epimastigotes/μl and the corresponding mortality is indicated by bars. (B) Representative flow cytometry pseudocolor charts are provided to illustrate the morphometric profile (Forward Scatter—Size vs Side Scatter—Granularity) and fluorescent pattern observed at control samples (incubated in the presence of PBS) as well as PI+FDA stained parasites and FDA single positive viable parasites. (TIFF) [file pntd.0003646.s001.tiff]
